# Saunders’ Question Compends—Essentials of Histology

**Published:** 1901-10

**Authors:** 


### Books and Magazines.

SAUNDERS' QUESTION COMPENDS—ESSENTIALS OF HISTOLOGY. By Louis Leroy, B. S., M. D., Professor of Pathology in Vanderbilt University; City and State Bacteriologist, Etc., Nashville, Tenn. Seventy-two illustrations. Pages, 231. Price, $1.00. Philadelphia: W. B. Saunders & Co., London, 161 Strand W. C. 1901.

The publishers of these little books say that over 175,000 copies have been sold since the first volume was issued. This proves that carefully condensed literature is popular with students and practitioners of medicine. Containing, as this book does, the essential facts in histology, a busy practitioner can review the subject without a waste of time. T. J. B.

